# A Lympho-Follicular Microenvironment Is Required for Pathological Prion Protein Deposition in Chronically Inflamed Tissues from Scrapie-Affected Sheep

**DOI:** 10.1371/journal.pone.0062830

**Published:** 2013-05-03

**Authors:** Caterina Maestrale, Giovanni Di Guardo, Maria Giovanna Cancedda, Giuseppe Marruchella, Mariangela Masia, Stefania Sechi, Simonetta Macciocu, Cinzia Santucciu, Mara Petruzzi, Ciriaco Ligios

**Affiliations:** 1 Dipartimento di Sanità Animale, Istituto Zooprofilattico Sperimentale della Sardegna, Sassari, Italy; 2 Dipartimento di Scienze Biomediche Comparate, Facoltà di Medicina Veterinaria, Università degli Studi di Teramo, Teramo, Italy; 3 Research Unit of Genetics and Biotechnology, DIRPA, AGRIS, Olmedo, Italy; Colorado State University, College of Veterinary Medicine and Biomedical Sciences, United States of America

## Abstract

In sheep scrapie, pathological prion protein (PrP^Sc^) deposition occurs in the lymphoreticular and central nervous systems. We investigated PrP^Sc^ distribution in scrapie-affected sheep showing simultaneous evidence of chronic lymphofollicular, lymphoproliferative/non-lymphofollicular, and/or granulomatous inflammations in their mammary gland, lung, and ileum. To do this, PrP^Sc^ detection was carried out via immunohistochemistry and Western Blotting techniques, as well as through inflammatory cell immunophenotyping. Expression studies of gene coding for biological factors modulating the host’s inflammatory response were also carried out. We demonstrated that ectopic PrP^Sc^ deposition occurs exclusively in the context of lymphofollicular inflammatory sites, inside newly formed and well-organized lymphoid follicles harboring follicular dendritic cells. On the contrary, no PrP^Sc^ deposition was detected in granulomas, even when they were closely located to newly formed lymphoid follicles. A significantly more consistent expression of lymphotoxin α and β mRNA was detected in lymphofollicular inflammation compared to the other two types, with lymphotoxin α and β signaling new lymphoid follicles’ formation and, likely, the occurrence of ectopic PrP^Sc^ deposition inside them. Our findings suggest that, in sheep co-affected by scrapie and chronic inflammatory conditions, only newly formed lymphoid follicles provide a suitable micro-environment that supports the scrapie agent’s replication in inflammatory sites, with an increased risk of prion shedding through body secretions/excretions.

## Introduction

Sheep and goat scrapie is the prototype of prion diseases, or transmissible spongiform encephalopathies (TSEs), a group of fatal neurodegenerative disorders that include bovine spongiform encephalopathy (BSE), chronic wasting disease (CWD) of deer, Creutzfeldt-Jakob disease (CJD), and variant Creutzfeldt-Jakob disease (vCJD) in humans [1].

The most prominent feature of TSEs is the deposition, both within the central nervous system (CNS) and in lymphoreticular system (LRS) tissues, of the pathological or disease-associated isoform (PrP^Sc^) of the normal, host-encoded cellular prion protein (PrP^C^) [1].

Most of the current understanding of TSE pathogenesis has been derived from sheep and mice studies. In both animal models, it has been demonstrated that PrP^Sc^ deposition is detectable within LRS tissues long before it is displayed at the CNS level. In this respect, ileal Peyer’s patches (PPs) are recognized as the earliest LRS site to be colonized by the scrapie agent [2], with follicular dendritic cells (FDCs) residing inside their germinal centres (GCs) and playing a crucial role in PrP^Sc^ replication [3].

Furthermore, although a wide PrP^Sc^ dissemination occurs in both extra-neural and non-LRS tissues such as skeletal muscle, placenta, and salivary glands in scrapie-affected sheep [4]–[6], it has also been shown that chronic inflammatory conditions may lead to PrP^Sc^ deposition in a range of additional tissue districts in TSE- affected animals. Such an intriguing phenomenon has been shown for the first time in transgenic mice harboring either lymphoproliferative inflammatory foci in their kidneys and pancreas [7], or granulomatous subcutaneous lesions [8]. In a similar manner, sheep affected by naturally occurring or experimentally induced scrapie, as well as CWD-affected deer, have displayed PrP^Sc^ deposition in mammary glands [9], [10] and kidneys [11] with chronic lymphoproliferative inflammation.

During chronic inflammation, both the etiological agent and the host’s immune response are known to drive the recruitment as well as the correct positioning and timing of the different immune cells. Tissues affected by chronic lymphoproliferative inflammation display variable numbers of T and B lymphocytes, along with plasma cells, macrophages, dendritic cells (DCs), and FDCs. All these inflammatory cells may frequently evolve into newly formed follicles similar to those found in secondary lymphoid organs, thus giving rise to a lymphofollicular inflammation pattern [12]. There are several human pathological conditions in which newly formed ectopic lymphoid follicles have been reported, including rheumatoid arthritis, Hashimoto’s thyroiditis, multiple sclerosis, and *Helicobacter pylori*-induced gastritis [13]–[16]. Similarly, the Maedi Visna virus (MVV), a cosmopolitan sheep retrovirus, causes chronic lymphoproliferative changes, which may frequently evolve into a lymphofollicular inflammation pattern at pulmonary and mammary gland levels [17].

Studies in mutant mice have established that the formation of lymphofollicular structures at inflammatory sites involves immune cell recruitment and compartmentalization. These processes are initially promoted by a subset of cytokines of the tumor-necrosis factor (TNF) family including TNFα, mainly expressed by lymphoid cells, mast cells, and endothelial cells, as well as by lymphotoxins α (LTα) and β (LTβ), chiefly expressed by CD79^+^ B and CD3^+^ T lymphocytes [18].

Also involved in the signalling cascade are a set of adhesion molecules such as vascular cell-adhesion molecule 1 (VCAM-1), along with a set of chemokines like cysteine-cysteine chemokine ligands 19 and 21 (CCL19, CCL21), as well as C-X-C motif chemokine ligands 12 and 13 (CXCL12, CXCL13) [12], [16], [18], [19].

Apart from the lymphofollicular pattern, chronic inflammation may also develop into another distinctive pattern known as granuloma. In this case, activated macrophages evolve into epithelioid or multinucleate giant cells and cooperate with T lymphocytes and stromal cells, thus forming well-defined fibro-cellular nodules. Granulomatous inflammations are very common in both domestic and wild animals affected by a number of bacterial, parasitic, and viral infections. The most common agents of granulomatous inflammation in sheep include *Mycobacterium avium* subsp. *paratuberculosis* (MAP), which causes an endemic granulomatous enteritis (paratuberculosis), and nematode parasites, which cause chronic bronchopneumonia characterized by the occurrence of granulomatous and/or lymphoproliferative lesions. Granulomas may also be sporadically caused by foreign bodies or inappropriate use of medication [20].

In organizing granulomatous inflammation, the host’s response appears to be characterized by the presence of γ-interferon producing T lymphocytes and the coexisting production of TNFα on behalf of macrophages. Additionally, some of the chemokines involved in lymphoneogenesis are also known to play a role in immune cell attraction and positioning within granulomas [21].

Thus far, ectopic PrP^Sc^ deposition in natural TSE host species has been shown to occur only at the level of lymphoproliferative inflammatory sites [9], [10]. However, the precise microscopic and biomolecular features of these inflammatory lesions shaping a micro-environment favorable for PrP^Sc^ deposition are far from being understood.

Consequently, this study was aimed at characterizing the micro-environment of chronic inflammatory sites in which PrP^Sc^ accumulates from a morphological and molecular standpoint. To do this, we conducted an innovative investigation on sheep co-affected by scrapie and by various forms of chronic inflammation. We focused upon the cellular components and expression patterns of the genes coding for the main cytokines, chemokine receptors, and a set of adhesion molecules involved in the signaling cascade of lymphoproliferative and granulomatous inflammatory responses.

## Materials and Methods

### Ethics Statement

The protocol used for slaughtering the animal subjects was officially approved by the Service for Animal Welfare of the Istituto Zooprofilattico Sperimentale of Sardinia (IZSS, Sassari, Italy) in accordance with guidelines n. I 09 044 and in tight agreement with the prescriptions of Italian National Law n. 116/1992. Slaughtering procedures were performed at the diagnostic laboratories of IZSS. Sheep owners gave permission for the animals to be used in the study.

### Selection of the Sheep

During the period 2005–2011, within the framework of the scrapie diagnostic surveillance activity carried out by IZSS, we selected 27 Sarda breed sheep simultaneously affected by scrapie and by other diseases characterized by chronic inflammation. All the sheep under study, which were collected from various farms located in Sardinia, carried at PrP gene codons 136, 154, and 171 ARQ/ARQ homozygous genotype, which has been associated with scrapie susceptibility in some ovine breeds, including Sarda sheep. Sheep were referred to the diagnostic laboratories of IZSS for a detailed *post mortem* examination and subsequent laboratory investigation. Firstly, sheep were humanely euthanized at the terminal stage of clinical scrapie with a barbiturate solution, followed by injection of 4 ml of embutramide and mebenzonic-iodide (Hoechst Roussel Vet, Italy). Next, we performed proper nervous and LRS tissue sampling (with tissues being both frozen at −80°C and fixed in 10% neutral buffered formalin) for scrapie diagnosis, which was carried out following a protocol developed by the *Italian National Reference Centre for Animal Encephalopathies* (CEA, Turin, Italy). Furthermore, all organs displaying macroscopic changes indicative of chronic inflammation were sampled. To do this, the diseased area of each organ was removed and subsequently divided into two parts, one of which was fixed in 10% neutral buffered formalin and the other stored at −80°C. All formalin-fixed tissues were then embedded in paraffin following routine laboratory protocols.

The causative agents of chronic inflammation were identified by applying suitable histochemical methods such as Ziehl-Neelsen (ZN) stain, along with serology and Real Time PCR (RT-PCR) investigation techniques. Details of the sheep included in the study are shown in Table 1.

**Table 1:** Details of the 27 sheep with clinical scrapie and chronic inflammation affecting a number of organs.

No. of cases	Organ	Causative agent	Histopathological pattern	PrP^Sc^ deposition +/−
5	Lung	Nematode parasites	Lymphofollicular	2/3
3	Lung	MVV	Lymphofollicular	2/1
3	Lung	Nematode parasites	Granulomatous and lymphofollicular	1*/2
2	Ileum	MAP	Granulomatous	0/2
7	Mammary gland	MVV	Lymphofollicular	5/2
4	Mammary gland	MVV	Lymphoproliferative/non-lymphofollicular	0/4
3	Mammary gland	Foreign body	Granulomatous	0/3

Results of PrP^Sc^ IHC at the sites of inflammation are also shown. *PrP^Sc^ deposition was seen occurring only within newly formed lymphoid follicles, either adjacent or not to granulomatous inflammatory lesions.

### Histopathology and PrP^Sc^ Immunohistochemistry

For histopathology, the paraffin-embedded tissue blocks were cut into 5 µm-thick sections, stained with hematoxylin and eosin (H&E), and subsequently examined under a light microscope. For PrP^Sc^ immunohistochemistry (IHC), 4 µm-thick sections, mounted on positively charged glass slides, were first dried overnight at 37°C, rehydrated, and then autoclaved at 121°C for 20 min in a solution of 0.01 M citric acid (pH 6.1), in order to better expose the antigen epitopes. Slides were then immersed in a solution of 0.3% H_2_O_2_ for 10 min at room temperature to quench endogenous peroxidase activity, rinsed with PBS, and non-specific sites were blocked in 5% BSA in PBS. Further steps included utilization of a biotin-streptavidin detection method (Vector Laboratories, Inc., USA.), using F99 (VMRD Inc., USA) (1∶800) as primary anti-PrP monoclonal antibody (MoAb). This MoAb is known to recognize a conserved epitope corresponding to a 220–225 peptide region of the ovine PrP sequence. Immune reactions were visualized following 3-3′-diaminobenzidine (DAB) chromogen solution (Dako, Denmark). Sections from organs and tissues of scrapie-uninfected sheep, which were found to harbor inflammatory changes similar to those observed in the scrapie-affected sheep under study, served as negative controls. Two additional negative control series were set up by omitting the primary MoAb, as well as by challenging similar tissues from sheep affected by unrelated pathologies. In the two sheep affected by scrapie and paratuberculosis, sections of ileum were first stained with the above mentioned PrP^Sc^ IHC technique and then subjected to a routine Ziehl-Neelsen (ZN) staining protocol in order to allow the simultaneous detection of PrP^Sc^ aggregates and MAP within the same sections.

### PrP^Sc^ Western Blotting

Frozen nervous, LRS, and mammary gland tissues were thawed and then subjected to an appropriate Western Blotting (WB) analysis for PrP^Sc^ detection. This was carried out by means of a modified Prionics check protocol (Prionics AG, Switzerland), with 0.5 g of mammary gland tissue being first incubated in TBS buffer (10 mM Tris HCl, 133 mM NaCl, pH 7.4) containing 1.5 mM CaCl_2_ and 2.5 mg/ml (final concentration) of type XI collagenase 1.6 U/mg (Sigma, USA) for 2 h at 37°C. Samples were then centrifuged at 30,000 g for 2–30′ at 4°C, with pellets being re-suspended in the homogenization buffer (20% w/v) which is included in the Prionics check kit, and homogenized using an automated system. A 5,000 µl volume of this homogenate was incubated with 500 µl of 10X digestion buffer and proteinase K (50 µg/ml), both of which were also included in the Prionics check kit, at 50°C for 40 min. The reaction was blocked with 50 µl of digestion stop buffer. After digestion, further concentration steps were performed by centrifugation at 30,000 g for 30′ at 4°C. Pellets were re-suspended in 20 µl of 1X NuPage SDS reducing buffer and 10 µl of each solution was loaded on 12% NuPAGE Novex Bis-Tris Gels 12 wells (Invitrogen, USA) for electrophoresis under constant application of 120 V for 45′. Electroblotting was performed onto polyvinylidene fluoride membranes (150 V for 1 h). For PrP^Sc^ detection, membranes were incubated overnight at 4°C with P4 MoAb (1∶15,000), which specifically recognizes the aa89–104 epitope of the ovine PrP sequence **(**R-Biopharm AG, Germany). After washing with a solution of 10 mM Tris HCl, 133 mM NaCl, and 0.2% Tween 20 (TBST), the secondary Ab diluted 1∶5000 was added to the reaction, with membranes being subsequently washed 4 times for 5′, equilibrated in luminescence buffer, placed in chemiluminescent substrate (Roche, Switzerland), and finally exposed to an X-Ray film. Examination of the *obex* and lymphoid tissue was carried out using the Prionics check kit, in accordance with the manufacturer’s instructions.

### Immunophenotypic Characterization of Inflammatory Cells

The *in situ* identification of macrophages, T and B lymphocytes, DCs, and FDCs was performed by detecting the cluster of differentiation (CD) antigen system. Specifically, CD172a and CD68 were used to identify macrophages, while CD11c was employed as DC marker. Furthermore, CD79, CD3, and CNA-42 were utilized for B cells, T lymphocytes, and FDCs, respectively. Briefly, 5–7 µm thick sections were obtained from the mammary gland, lung, and ileum, to be subsequently placed on polilysine-L-coated glass slides for paraffin wax removal with xylene and rehydration in an ethanol series.

For CD172a, CD68, and CD11c antigen retrieval, we used a microwave oven at 850 W (3 runs each of 5′), with sections being immersed in EDTA (10 mM) at pH 8. CNA-42 antigen retrieval was also carried out by microwave oven at 850 W (3 runs each of 5′) in sodium citrate buffer (pH 6.0). Finally, sections were immersed in pH 9 Tris Buffer EDTA solution and treated two times in a microwave oven at 850 W for 10′for CD3 and CD79 antigen retrieval.

For FDC identification, sections were immersed in DakoCytomation Target Retrieval pH 9.9 solution (Dako, Demark) at 97.8°C for 15′, while CD172a antigen retrieval was carried out by immersing sections in 99% 0.1 M formic acid and treating them with a microwave oven at 600 W for 15′. Against the CD antigens under investigation we used the following primary antibodies: anti-bovine CD172a MoAb (VMRD Inc, USA), anti-human CD79 MoAb (DakoCytomation, Denmark), anti-human CD3 polyclonal Ab (DakoCytomation, Denmark), anti-human CNA-42 MoAb (DakoCytomation, Denmark), anti-human CD68 MoAb (AbD Serotec, United Kingdom), and anti-human CD11c MoAb (Abcam, USA).

The subsequent steps of the protocol were similar to those used for PrP^Sc^ IHC. Furthermore, in order to comparatively evaluate immune cell positioning in normal secondary lymphoid follicles and in newly developed lymphoid follicles, palatine tonsil sections from all the sheep with chronic lymphofollicular inflammatory lesions included in the present study were investigated by detecting the same CD antigens. Suitable sections from scrapie-free sheep were used as CD-positive controls. Negative controls included sections stained by omitting the primary antibody, with subsequent staining only with a secondary antibody or unrelated rabbit serum.

### Gene Expression Profiles in Mammary Glands

The relative Real Time PCR quantification (qRT-PCR) was performed only on sheep mammary glands in which chronic inflammatory lesions were found, as well as on microbiologically and histologically healthy mammary glands from 6 scrapie-free sheep belonging to the same flocks. The gene transcripts of LTα, LTβ, and TNFα cytokines, CCL19, CCL21, CXCL12, and CXCL13 chemokines, as well as their respective receptors LTβR (for LTα and LTβ), TNFR1 (for TNFα), CCR7 (for CCL19 and CCL21), and CXCR4 (for CXCL12 and CXCL13), together with lymphatic vessel endothelial hyaluronan receptor-1 (LYVE-1) and vascular cell adhesion molecule 1 (VCAM-1) were quantified. The complete genomic sequence of the each mRNA was performed by collecting, from different databases, short sequences of *Ovis aries* which were assembled by using CLC Genomic Workbanch software. Genomic sequences of *Bos taurus* were used as reference genes. On the same mammary glands, PrP gene expression was also quantified. In this respect, primers were designed to the corresponding ovine PrP gene sequences (GenBank number: AF195247).

Details of all the primers used for qRT-PCR are reported in Table 2. Total RNA was isolated from 25 mg of mammary gland parenchyma with a RNeasy Fibrous Tissues mini kit (Qiagen, USA), following the manufacturer’s instructions. Genomic DNA contamination was kept to a minimum including a DNA digestion step (DNAse Qiagen, USA).

**Table 2:** Details of the primers used to determine cytokine, chemokine, and adhesion molecule gene expression levels.

Gene	Primer sequences and orientation (5′ to 3′)	MGB-FAM probe
**LTα**	**Fwd-**ATG GCT TCT CTC TCA GCA ACA A **; Rev-**AGA CCA CTT GGG AGT AGA CGA A	CTC CCT CCT GGT CCC C
**LTβ**	**Fwd-**AGC GGG ACG CTG TTC TC **; Rev-**CCG ACG TGA CAG TAG AGG TAA TAG A	CAG CCC TTC CGC ACC C
**LTBR**	**Fwd-**CAA AGA TGA AGT CAC GGA GGC TAA; **Rev-**AGA GGA GGT GTT CTG GAA GTG T	CAG CAA CTG TGT CCC C
**TNFα**	**Fwd-**CGT GGA GCT GAA AGA CAA CCA; **Rev-**AGA GGA CCT GCG AGT AGA TGA G	CAC TGA CGG GCT TTA C
**TNFR1**	**Fwd-**CTG GTG ATT GTC TTT GGG CTT T; **Rev-**AGC GAC ATG CTA AAA CCA CAG A	CCT GGC ATC CTT CGC C
**CXCL12**	**Fwd-**GTC GCC AAA GCC AAC GT; **Rev-**GGG CAC AGT TTG GAG TGT TGA	AAG CAC CTC AAG ATC C
**CXCR4**	**Fwd-**CCA TGC CAC CAA CAG TCA GA; **Rev-**CAG ACA CCA ACA TAG ACC ACC TT	TTC AGC CAG CAG CTT C
**CXCL13**	**Fwd-**CTG GAG CTC TGC TTC TTA TGC T; **Rev-**CAG AAC ACC GTG GAC AGG AG	CTG GCC TTC AGC CTC T
**CXCR5**	**Fwd-**GTC AGT GAA CCC CAT TAC AAC GA; **Rev-**CAT TGG TTT CGG CTTGGT TCT C	CTC CCT GCC ACA CTG C
**CCL19**	**Fwd-**CGC CCC ATC CCT GTA TTC C; **Rev-**CAG CCT GCA GCC ATC CT	CCG CTA CCT GCT CCT C
**CCL21**	**Fwd-**AGG CCT GGG TGC AGA AC; **Rev-**CAG TCC TGG GCT GGT TTC C	CCAGACGCCGCATCAG
**CCR7**	**Fwd-**CTG GAT GCT GGC CAT GGT; **Rev-**TCT TCT GGA TCC CGC TGT ACA T	CTC CAC CCC AGA GCT G
**LYVE1**	**Fwd-**ACT TGG ATT AAC TCA TGC TTT CCA GAA; **Rev-**GTG TAT GTT TCA GTT TCA GCG TTG A	CAC CAC CGA TGA CCC C
**VCAM1**	**Fwd-**CCA GAT AGA CAG CCC TCT GAA C; **Rev-** CAG GGT CAA CAT GGA CTT GGA	CTC GCT CCT CAC CTT C
**PrP**	**Fwd–**TGG CAA TGA CTA TGA GGA CCG; **Rev-** TGG TCT GTA GTA CAC TTG GTT GGG	ACT ATC GTG AAA ACA T
**18S** *	**Fwd-**CGG AAC TGA GGC CAT GAT TAA GAG; **Rev-** TTC GCT CTG GTC CGT CTT G	TCT AGC GGC GCA ATA C

* Sheep 18S gene was used as housekeeping control gene.

Since there was no risk of interference by other cellular components, the isolation of pure poly A mRNA was made from 5 µg of total RNA until a total 40 µl of volume was reached by using an Oligotex mRNA kit (Qiagen, USA).

The reverse transcription of mRNA was performed by using a Quantitect Reverse Transcription Kit (Qiagen, USA) with oligodT from 12 µl of pure poly A mRNA until a total 20 µl of volume was reached.

Gene expression assays were designed by Assays-by-Design for Gene Expression Assays (Life Technologies, USA) by using TaqMan FAM dye-labelled MGB probes. Gene expression quantification using these assays was performed on ABI PRISM 7900HT Sequence Detection System (Life Technologies, USA) utilizing the universal thermal cycling parameters of the instrument. Gene expression levels were determined by making reference to the housekeeping sheep 18S gene (Assays-by-Design for Gene Expression Assays, Life Technologies, USA).

Data was analyzed using the Sequence Detection Systems 2.3 software (Life Technologies, USA).

The relative cytokine, chemokine, and adhesion gene expression levels were calculated using the previously described 2^−ΔΔCT^ method. Data was expressed as mean ± SD.

### PrP Quantification

The total PrP amount was determined in the same mammary glands which were examined for PrP gene expression quantification. Firstly, we quantified the total tissue proteins in both healthy and inflamed mammary glands by using the BCA Protein Assay Kit (23–225 BCA™ Protein Assay Kit, Pierce, USA). Mammary gland samples were normalized in relation to protein amount and then subjected to the ELISA TeSeE test (Bio-Rad, USA) for PrP detection after omitting the proteinase K (pK) treatment. The means of the optical density (OD) signals detected in each group of mammary glands were used to indicate the relative PrP amounts in both inflamed and healthy mammary glands.

### Statistical Analysis

Cytokine, chemokine, receptor, and adhesion gene expression levels were Log10(x+1) transformed in order to normalize the distribution. The log-transformed data was compared using a general linear model. Tukey’s test for multiple comparisons was used to compare the results across the 4 different groups of sheep under study. P-values <0.05 were considered to be statistically significant. All statistical analyses were performed with R version 2.15.0 (R Foundation for Statistical Computing, Austria).

## Results

### Pathology

**Figure 1:**
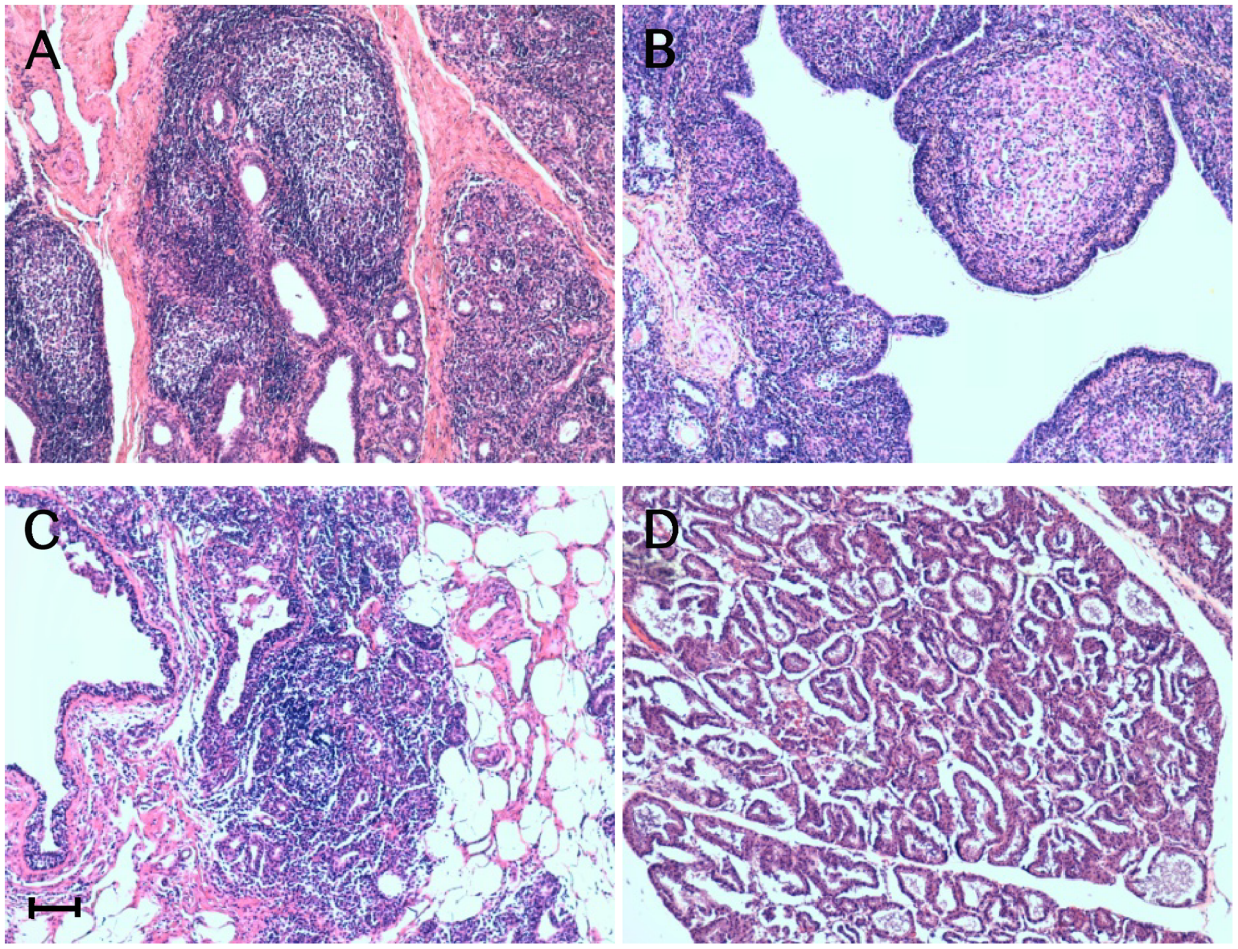
Inflammatory lesions patterns in ovine mammary glands. Micrographs show the inflammatory lesions’ morphology in mammary glands from sheep concurrently affected by scrapie and mastitis. Representative patterns of lymphofollicular (A), granulomatous (B), and lymphoproliferative/non-lymphofollicular (C) mastitis. A histologically normal ovine mammary gland is also shown (D). Hematoxylin-eosin (H&E) stain. Scale bar = 100 µm.

Gross and histopathological investigations yielded a total number of 27 sheep affected by scrapie and associated lesions indicative of chronic inflammation in mammary glands, lungs, and ileum (Table 1). Histologically, chronically inflamed mammary glands showed 3 distinct morphological lesion patterns, which were classified as lymphofollicular, lymphoproliferative/non-lymphofollicular, and granulomatous mastitis (Figure 1). The first two lesional patterns indicated MVV as their cause, with this viral agent being detected by RT-PCR. Furthermore, in all cases of lymphoproliferative/non-lymphofollicular mastitis, we observed variable numbers of lymphocytes and plasma cells infiltrating, along with a few macrophages, the interlobular and interalveolar spaces. As far as the lymphofollicular lesion pattern is specifically concerned, this was characterized by a number of lymphoid cell aggregates containing GCs resembling secondary follicles.

Granulomatous mastitis appeared to be the consequence of a foreign body reaction after inappropriate intramammary injection of an antibiotic solution. Microscopically, all these cases exhibited coalescing granulomas formed by clusters of macrophages, epithelioid cells, and lymphocytes, along with a fibroblastic reaction (Figure 1).

**Figure 2:**
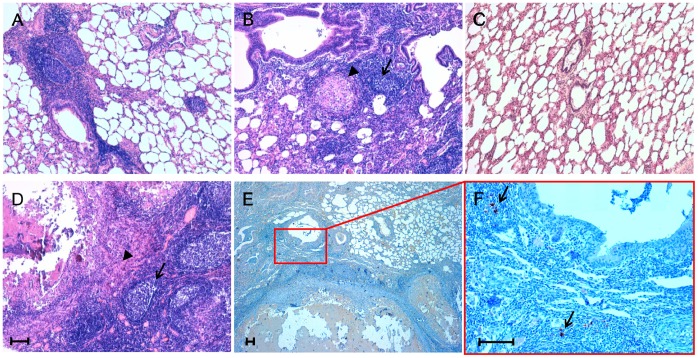
Inflammatory lesion patterns and PrP^Sc^ deposition in ovine lungs. Micrographs show the inflammatory lesions’ morphology and PrP^Sc^ deposition in lungs from sheep simultaneously affected by scrapie and parasitic bronchopneumonia caused by nematodes. Representative patterns of lymphofollicular (A) and lymphofollicular (arrows) associated with granulomatous (arrowheads) inflammation (B and D). A histologically normal ovine lung is also shown (C). PrP^Sc^ deposits (arrows) are visible within newly formed lymphoid follicles adjacent to granulomatous lesions (E and F). Micrograph F is a higher magnification of the red line-enclosed area shown in micrograph E. Hematoxylin-eosin (H&E) stain (A, B, C, and D); PrP^Sc^ immunohistochemistry (IHC) with F99 as primary antibody and Mayer’s hematoxylin counterstain (E and F). Scale bar = 100 µm.

In the lung tissue from 8 out of the 27 sheep included in this study (Table 1), we detected pale or dark subpleural nodules 2–15 mm in diameter, which were mainly seen affecting the caudal lobes. These areas were consistent with inflammatory foci caused by different species of nematode parasites, such as *Muellerius* spp. and *Cystocaulus* spp., two lungworms of sheep which are commonly found in both larval and adult forms within bronchiolar and alveolar *lumina*. Histologically, we observed focal or multifocal interstitial pneumonia characterized by lymphoid cell infiltrates, along with a variable number of newly formed lymphoid follicles. Additionally, in 3 out of the 8 sheep with pneumonic lesions, well-organized fibro-cellular granulomas containing multinucleate giant cells, epithelioid cells, macrophages, and lymphocytes were seen in close association with newly formed lymphoid follicles (Figure 2).

**Figure 3:**
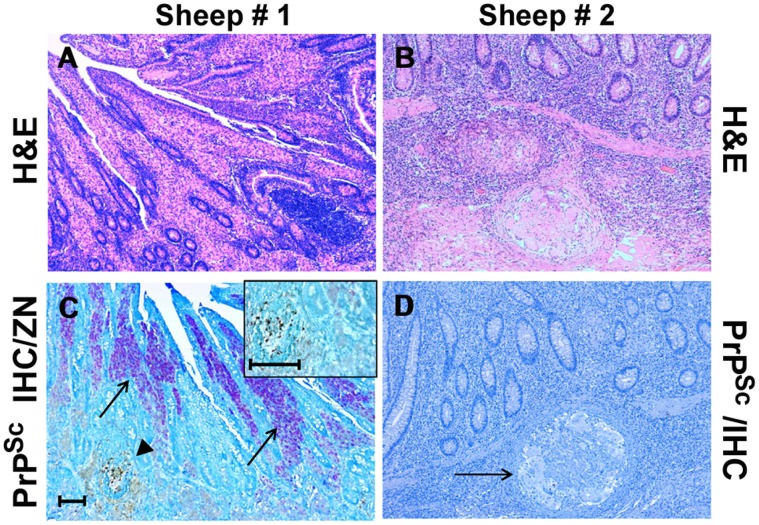
Inflammatory lesion patterns and PrP^Sc^ deposition in the ileum of sheep with scrapie and paratuberculosis. Sheep #1: Granulomatous enteritis is shown, with inflammatory gut lesions exhibiting a “lepromatous” morphology (A); by means of IHC, PrP^Sc^ deposition is apparent inside a constitutive lymphoid follicle of ileal Peyer’s patches (arrowhead), while no PrP^Sc^-positive immunostaining is detectable within the surrounding granulomatous inflammatory foci, in which consistent numbers of acid-fast bacilli (*M. avium* subsp. *paratuberculosis*, MAP) are present inside epithelioid macrophages (arrows); a higher magnification of the above lymphoid follicle, harboring PrP^Sc^ aggregates, is shown in the inset (C). Sheep # 2: Granulomatous enteritis is shown, with inflammatory gut lesions exhibiting a “tuberculoid” morphology (B); no IHC evidence of PrP^Sc^ deposition is observed inside a microgranuloma (D, arrow). H&E stain (A-B); Ziehl-Neelsen stain and PrP^Sc^ IHC with F99 as primary antibody (C, see also “Materials and Methods”); PrP^Sc^ IHC with F99 as primary antibody and Mayer’s hematoxylin counterstain (D). Scale bars = 100 µm and 50 µm (inset of C).

Two out of the 27 sheep under investigation were affected by a severe transmural granulomatous ileitis suggestive of paratuberculosis, which was subsequently confirmed histochemically (ZN), biomolecularly (RT-PCR), and by means of serology against MAP. In the first of these two cases, lesions were characterized by a “lepromatous-type” morphology, with epithelioid cells containing numerous MAP organisms (multibacillary form), lymphocytes, and plasma cells infiltrating the *lamina propria*. In the second case, we observed numerous intramural “tuberculoid-like” granulomas with macrophages, multinucleate giant cells, and rare lymphocytes surrounded by an outer fibroblastic reactive wall (Figure 3). In this latter case, no MAP organisms were observed (paucibacillary form).

### Cell Immunophenotyping

**Figure 4:**
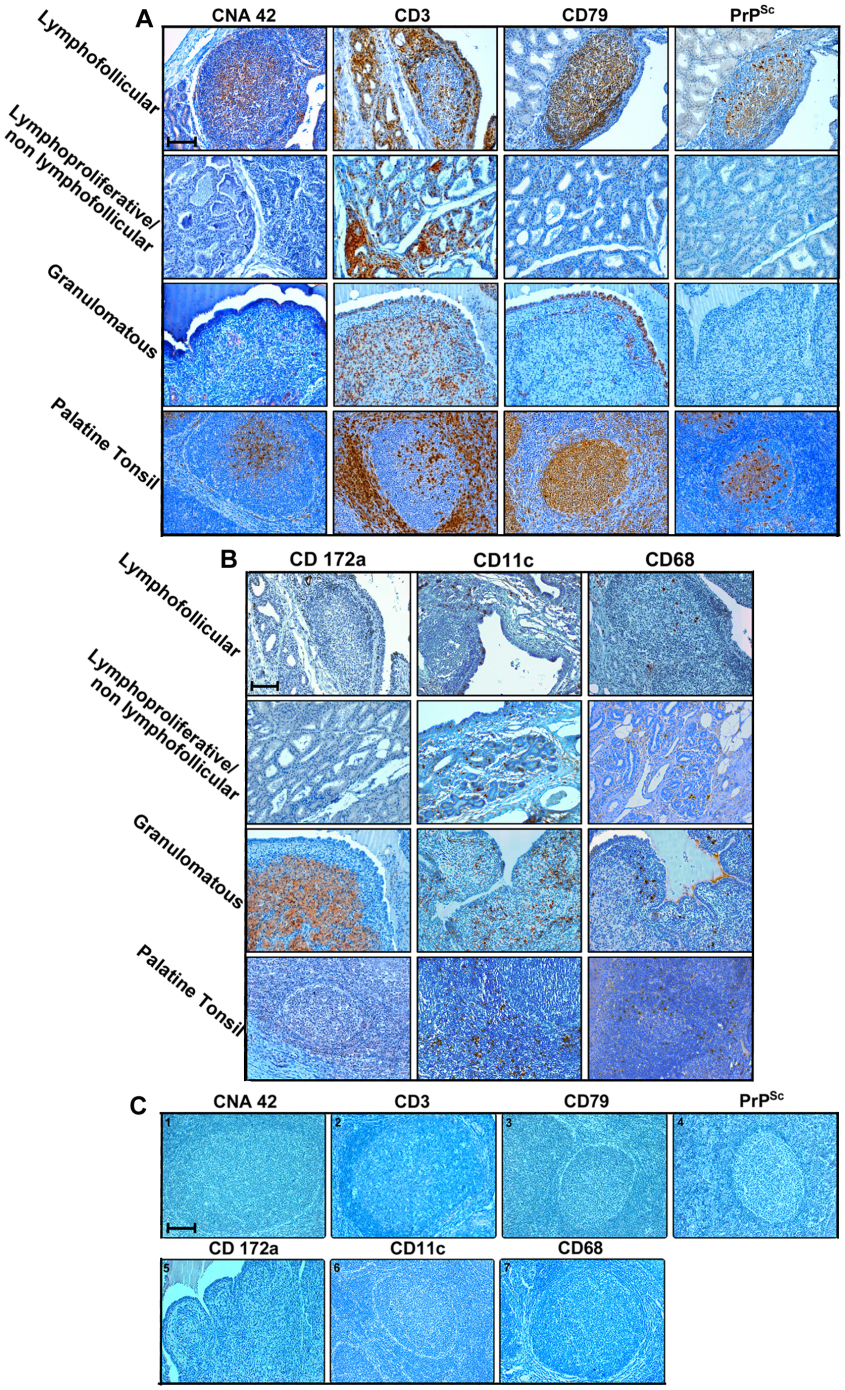
PrP^Sc^ is detected only in inflamed sheep mammary glands containing newly formed lymphoid follicles. Inflammatory cell immunophenotyping in serial paraffin-embedded sections of mammary glands from sheep simultaneously affected by scrapie and lymphofollicular, lymphoproliferative/non-lymphofollicular, or granulomatous mastitis. A normal ovine palatine tonsil is also shown for comparison. CNA-42, CD3, and CD79 were used as markers for FDCs, T cells, and B cells (Panel A), while CD172a, CD68, and CD11c were used for activated macrophages and DCs (Panel B), respectively. In panel C, a representative set of negative controls are shown. These were obtained by omitting all the primary antibodies used on the tonsil sections (photos 1, 2, 3, 4, 6, and 7), as well as from granulomatous mastitis (photo 5). The omitted primary antibodies are indicated on each photo. It is noteworthy that the relative positioning of these cell populations was similar in newly formed lymphoid follicles and constitutive follicles normally hosted inside the palatine tonsil. CD172a^+^ macrophages were the most representative cell population in granulomatous mastitis, which also displayed a moderate presence of T and B cells and DCs. CD3^+^ T cells were the most abundant cellular component in the context of lymphoproliferative/non-lymphofollicular mastitis. PrP^Sc^ deposition occurred in co-localization with CD79^+^ B cells and the CNA42^+^ FDC network both in palatine tonsil and in newly formed lymphoid follicles. Immune reactions were visualized by 3-3′-diaminobenzidine (DAB) chromogen, with F99 primary MoAb being used for PrP^Sc^ detection. Mayer’s hematoxylin counterstain. Scale bar = 100 µm.

The inflammatory cell immunophenotyping that was carried out on the mammary glands from all 14 sheep affected by chronic mastitis yielded the results outlined here below. Newly formed lymphofollicular structures were characterized by the presence of well-defined areas populated by CD79^+^ B lymphocytes forming GCs, which were surrounded by CD3^+^ T lymphocytes, along with CD11c^+^ DCs and rare CD68^+^ cells, as observed in perifollicular areas of the palatine tonsil (Figure 4A and 4B). Additionally, in serial mammary gland sections, GCs exhibited a CNA42^+^ FDC network. Therefore, the micro-architecture and organization of newly formed lymphoid follicles appeared to be strikingly similar to those of secondary lymphoid organs, as in palatine tonsils (Figure 4A and 4B). The lymphoproliferative/non-lymphofollicular infiltrate appeared to be mostly, if not totally, represented by CD3^+^ T lymphocytes, with scattered CD11c+ DCs and CD68+ cells (Figure 4A and 4B).

As far as granulomatous mastitis is concerned, we observed nodular cell aggregations close to milk ducts, mostly comprising CD172a^+^ epithelioid-like macrophages and showing very rare B lymphocytes. These were mostly located at the margin of granulomas, with scattered CD3^+^ T cells and CD11c+ DCs also apparent throughout the inflamed mammary tissue (Figure 4A and B).

In both sheep affected by MAP-associated granulomatous ileitis, CD172a+ epithelioid macrophages were the most prominent cell component, although rare T and B lymphocytes could also be observed.

### PrP^Sc^ Immunohistochemistry

PrP^Sc^ deposition was detected by IHC in both nervous and LRS tissues from all the clinical scrapie-affected animals included in the study.

Interestingly, IHC evidence of ectopic PrP^Sc^ deposition in chronic inflammatory sites was exclusively found in sheep co-affected by scrapie and lymphofollicular inflammation (Figures 4A and 4B), with no evidence of PrP^Sc^ accumulation being observed in any ovine co-affected by scrapie and lymproliferative/non-lymphofollicular inflammation, as well as by granulomatous inflammation (Figures 4A and 4B). Interestingly, in all the lung tissue samples in which newly formed follicles were strictly associated with granulomas, PrP^Sc^ aggregates were seen only inside the follicles (Figure 2). The PrP^Sc^ deposition pattern within newly formed lymphoid follicles was very similar to that observed in secondary lymphoid follicles from the same animals (Figure 4A). Furthermore, no evidence of PrP^Sc^ deposition was found in MAP-associated granulomatous lesions surrounding ileal PP follicles, independently from the occurrence of immunohistochemically detectable PrP^Sc^ aggregates inside the latter (Figure 3).

### PrP^Sc^ Western Blotting

PrP^Sc^ deposition was detected via WB analysis in both nervous and LRS tissues from all the clinical scrapie-affected animals included in the study.

**Figure 5:**
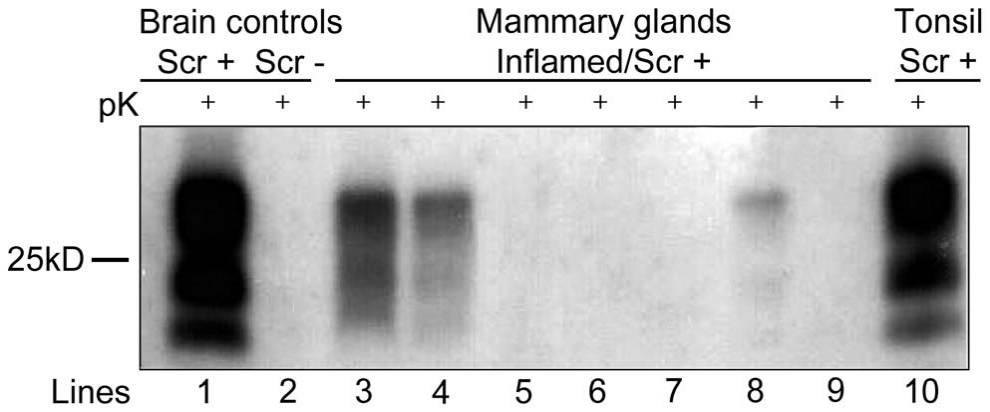
Western Blotting (WB) analysis for PrP^Sc^ in ovine mammary glands with chronic mastitis. Lines: 1–2 =  brain from a naturally scrapie-affected (Scr^+^) or scrapie-free (Scr^-^) sheep. Lines: 3–4–8 =  mammary glands from scrapie-affected sheep displaying lymphofollicular mastitis. Lines: 5–6–7 =  mammary glands from scrapie-affected sheep displaying granulomatous mastitis. Line 9 =  mammary gland from a scrapie-affected sheep displaying lymphoproliferative/non-lymphofollicular mastitis. Line 10 =  palatine tonsil from a naturally scrapie-affected sheep showing PrP^Sc^ in mammary gland with lymphofollicular mastitis. Among the mammary glands, positive WB signals were observed only in the case of lymphofollicular mastitis.

In this respect, considering that both the lung and the ileum have a constitutive “associated lymphoid tissue” which is not physiologically present within the mammary gland parenchyma, WB analysis at the mammary gland level was considered adequate for establishing an exclusive PrP^Sc^ deposition inside newly formed lymphoid follicles. Clear-cut evidence of PrP^Sc^ deposition was found in 5 out of 7 mammary glands affected by lymphofollicular mastitis, as also demonstrated by IHC. Furthermore, WB analysis did not detect any evidence of PrP^Sc^ deposition in the 3 sheep affected by granulomatous mastitis, still in agreement with IHC results (Table 1; Figure 5).

### Gene Expression Profiles

We also evaluated the expression levels of a number of genes which code for biological factors that are involved in the development of both lymphofollicular and granulomatous inflammation patterns. This was done only in sheep mammary glands in which chronic inflammatory lesions were found, as well as in microbiologically and histologically healthy mammary glands. Our decision to only analyze mammary glands for this purpose is justified by the fact that, unlike the lung or ileum, these structures display a physiological lack of any constitutive “associated lymphoid tissue”. Indeed, the presence of any constitutively “associated lymphoid tissue” would have altered the quantification of the expression levels of genes coding for the aforementioned biological factors.

On the basis of the results of our microbiological and histological investigations (Table 1), we identified the following 4 groups of sheep: 7 sheep with lymphofollicular mastitis (Group A); 4 sheep with lymphoproliferative/non-lymphofollicular mastitis (Group B); 3 sheep with granulomatous mastitis (Group C); 6 healthy control sheep (Group D).

The quantification of mRNA expression by RT-PCR obtained for each molecule represents the fold changes occurring in comparison to the histologically and microbiologically healthy mammary gland from a scrapie-free sheep that we used as the reference value.

**Figure 6:**
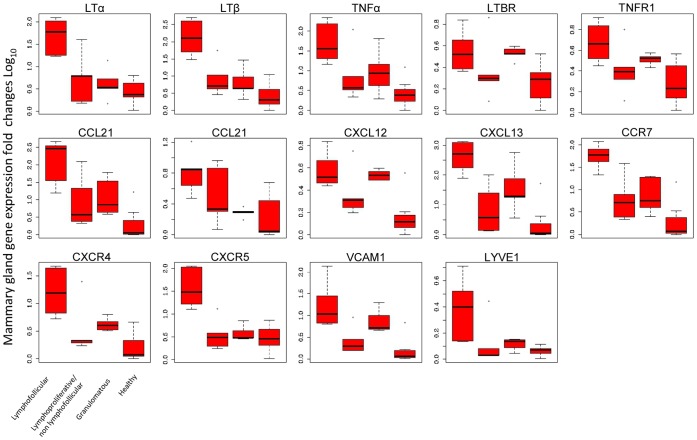
Differential qRT-PCR gene expression for cytokines, chemokines, receptors, and adhesion molecules involved in inflammatory responses. Total mRNA was isolated from inflamed sheep mammary glands showing lymphofollicular, lymphoproliferative/non-lymphofollicular, and granulomatous mastitis, as well as from histologically and microbiologically healthy ovine mammary glands (Groups A, B, C, and D, respectively, as described in “Materials and Methods”). Differences in specific gene expression levels were calculated as fold changes in transcripts between the mammary gland tissue samples under study *versus* one histologically and microbiologically healthy sheep mammary gland, which was used as the reference value. Each boxplot represents log-transformed gene expression levels in the different groups of cytokines, chemokines, and their receptors, together with adhesion molecules. The box represents the 25^th^ to 75^th^ quartile, the whiskers represent the range, and the horizontal line in the box is the median. White dots beyond the whiskers indicate outliers. The sample values were normalized to housekeeping sheep 18S gene level. The X-axis legend is common for all 14 graphics. *P* values among the different groups are shown in Table 3.

**Figure 7:**
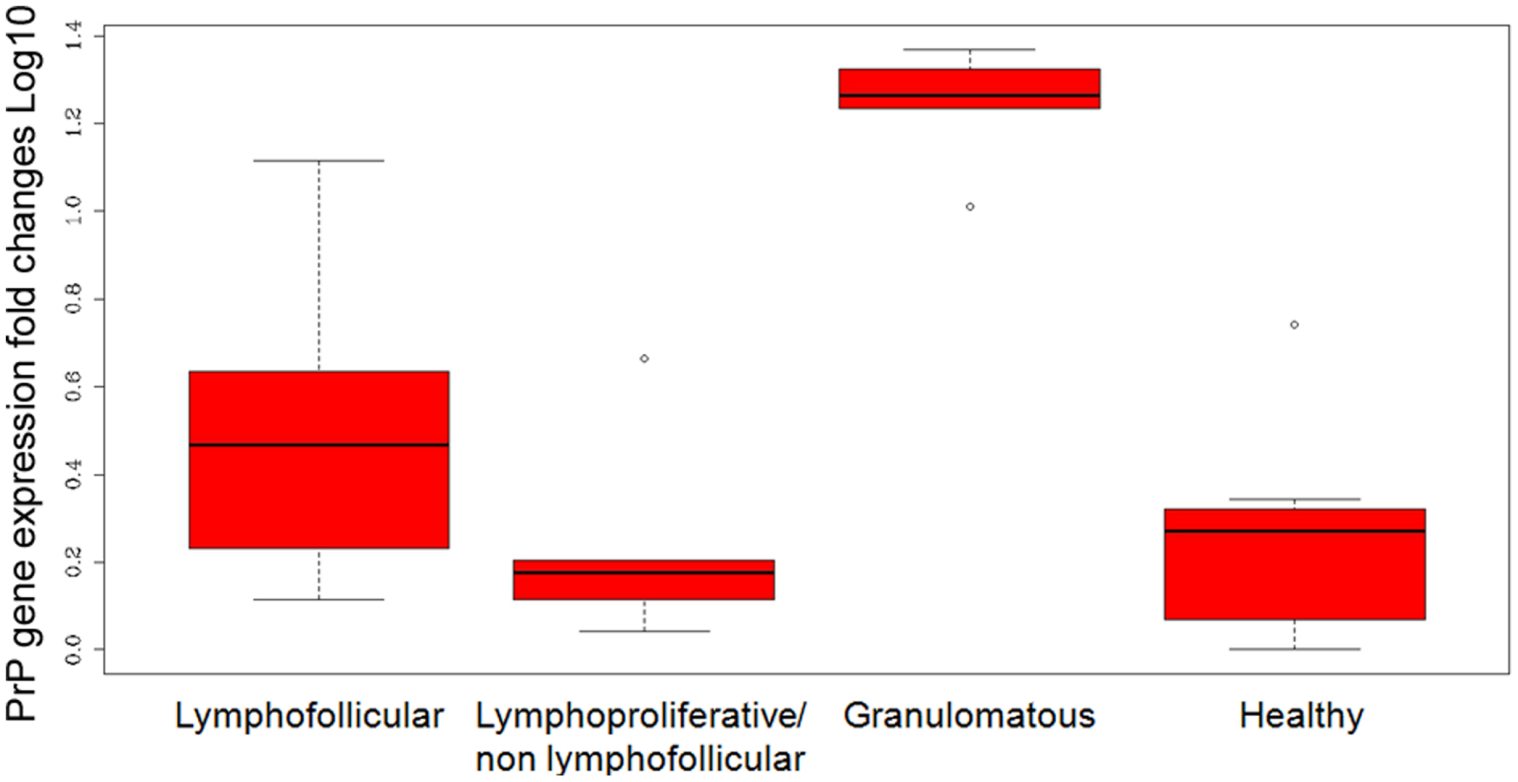
qRT-PCR expression of PrP gene in sheep mammary glands. Transcript levels are shown in mammary glands with lymphofollicular, lymphoproliferative/non-lymphofollicular, and granulomatous mastitis, as well as in histologically and microbiologically healthy ovine mammary glands (Groups A, B, C, and D, respectively, as described in “Materials and Methods”). Transcripts were normalized to housekeeping sheep 18S gene level. Differences in PrP gene expression levels were calculated as fold changes in transcripts between the mammary gland tissue samples under study *versus* one histologically and microbiologically healthy sheep mammary gland, which was used as the reference value. The box represents the 25^th^ to 75^th^ quartile, the whiskers represent the range, and the horizontal line in the box is the median. White dots beyond the whiskers indicate outliers. The sample values were normalized to housekeeping sheep 18S gene level.

For all the cytokines, chemokines, adhesion molecule, and receptors under study (with the exception of LTβR, which appeared to be similarly expressed in all groups), we observed significantly increased (P-values <0.05) levels of transcription in all mammary glands with lymphofollicular mastitis (Group A) compared to healthy mammary glands. In addition, mRNA expression was significantly more elevated for LTα, (P-values = 0.003 and 0.001), LTβ (P-values = 0.001 and 0.000), CCL19 (P-values = 0.016 and 0.036), CCR7 (P-values = 0.004 and 0.009), CXCR5 (P-value = 0.000), as well as for LYVE-1 (P-values = 0.039 and 0.33) in Group A *versus* Groups B, C, and D, respectively (Table 3). As far as Group C sheep are concerned, we observed that CXCL12 and VCAM-1 were significantly up-regulated in mammary glands affected by granulomatous mastitis as compared to healthy mammary glands (P-values = 0.005 and 0.022, respectively), demonstrating that some molecular determinants may still play an important role independently from inflammatory lesions’ morphopathological development. Nevertheless, the gene expression levels appeared to be similar among sheep with granulomatous and lymphoproliferative/non lymphofollicular mastitis (Table 3; Figure 6). Furthermore, PrP gene transcript levels were statistically higher in the mammary glands affected by granulomatous mastitis (P-values <0.001) in comparison to the other inflamed or healthy mammary glands (Figure 7). All details regarding gene expression quantification are shown in Table 3 and Figures 6–7.

**Table 3:** Cytokine, chemokine, receptor, adhesion molecule, and PrP gene expression levels.

Molecule	Histological pattern				P-value (P-value <0.05)*					
	Lymphofollicular	Lymphoproliferative/non-lymphofollicular	Granulomatous	Healthy	L vs LnL	L vs G	L vs H	LnL vs G	LnL vs H	G vs H
**LTα**	62.83±43.99	10.06±16.24	4.42±4.82	2.21±1.82	0.0031	0.0012	0.0001	0.9761	0.6406	0.8769
**LTβ**	207.03±198.98	14.56±22.34	9±11.36	2.62±3.51	0.0013	0.0006	0.0000	0.9845	0.2660	0.4530
**TNFα**	78.91±85.08	24.26±47.44	17.71±25.82	2.75±3.9	0.0711	0.1322	0.0017	0.9888	0.4973	0.3174
**LTBR**	2.8±1.73	1.9±2.47	2.41±0.47	0.92±0.81	0.4562	0.9988	0.0567	0.5756	0.7172	0.0991
**TNFR1**	4.16±2.26	1.98±1.93	2.28±0.4	1.14±1.05	0.1490	0.5234	0.0095	0.8462	0.6902	0.2273
**CCL19**	236.49±175.91	29.29±51.87	20.98±24.8	2.81±5.77	0.0160	0.0366	0.0001	0.9813	0.2878	0.1476
**CCL21**	6.46±4.59	3.37±3.63	0.95±0.26	1.2±1.75	0.3001	0.0270	0.0079	0.6057	0.9920	0.3872
**CXCL12**	2.96±1.55	1.62±1.69	2.45±0.38	0.61±0.9	0.1965	0.9811	0.0015	0.3881	0.2034	0.0059
**CXCL13**	634.69±522.43	25.41±42.71	134.97±240.91	7.81±19.04	0.0022	0.0792	0.0001	0.3986	0.0391	0.6356
**CCR7**	62.72±33.66	10.22±15.33	9.18±8.41	2.46±5.09	0.0046	0.0096	0.0000	0.9882	0.1955	0.1057
**CXCR4**	22.3±19.19	5.55±10.19	3.34±1.27	0.98±1.48	0.0213	0.0596	0.0004	0.9629	0.4747	0.2280
**CXCR5**	50.98±45.78	3.74±4.68	3.11±1.85	2.57±2.29	0.0003	0.0004	0.0000	0.9977	0.9783	0.9321
**VCAM1**	32.54±51.84	2.41±3.24	7.93±6.48	1.03±2.15	0.0094	0.4340	0.0004	0.2322	0.7084	0.0220
**LYVE1**	1.73±1.47	0.44±0.75	0.3±0.13	0.16±0.09	0.0398	0.0330	0.0048	0.9998	0.8999	0.9320
**PrP**	3.42±4.39	1.02±1.45	17.02±4.99	1.13±1.56	0.3832	0.0012	0.3636	0.0001	0.0000	0.9996

For the different groups the data are shown as mean values ± SD. Multiple comparisons were made with Tukey’s test.

* P<0.05 values were considered to be statistically significant.

L =  lymphofollicular; LnL =  lymphoproliferative/non-lymphofollicular; G =  granulomatous; H =  healthy.

### PrP Quantification

**Figure 8:**
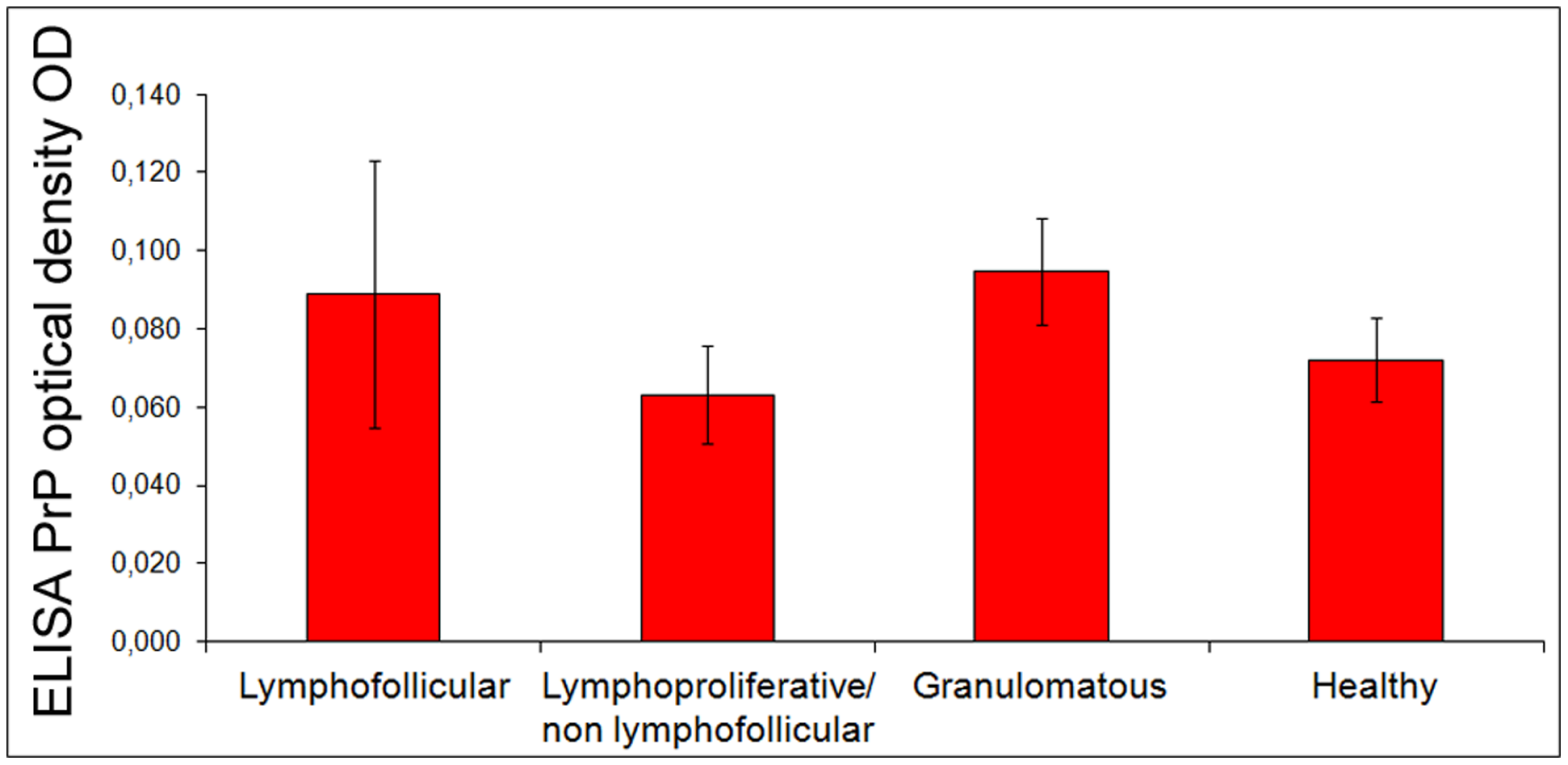
PrP quantification in ovine mammary glands. PrP is quantified in mammary glands with lymphofollicular, lymphoproliferative/non-lymphofollicular, and granulomatous mastitis, as well as in histologically and microbiologically healthy sheep mammary glands (Groups A, B, C, and D, respectively, as described in “Materials and Methods”). Differences among the 4 groups are represented as PrP optical density (OD) signals. The mammary gland samples were normalized in relation to the total protein amount.

Using an ELISA test, we were able to demonstrate the presence of variable PrP amounts among the groups of chronically inflamed and healthy mammary glands (Figure 8), with no statistically significant differences being found.

## Discussion

Although mammary glands from scrapie and MVV co-infected sheep were previously shown to allow ectopic PrP^Sc^ deposition inside lymphoproliferative mastitis lesions [9], we obtained clear-cut evidence that newly formed lymphoid follicles are essential for PrP^Sc^ deposition in chronically inflamed mammary glands and lungs from scrapie-affected sheep. We also found no evidence of PrP^Sc^ accumulation in mammary glands, lungs, and ileum of sheep simultaneously co-affected by scrapie and lymphoproliferative/non-lymphofollicular or granulomatous inflammation.

This appears to be in contrast with the results of a previous study, in which PrP^Sc^ aggregates were observed in experimentally induced subcutaneous granulomas of scrapie-infected transgenic mice [8]. In that study, PrP^Sc^ deposition occurred in mesenchymal cells, with fibroblastic reticular cells probably being most affected. The reasons behind such discordance remain uncertain. One possible explanation could be that the granulomatous lesions we investigated did not have the same etiology; our study also focused on a different species and different affected organs.

Our study clearly shows that the only factor driving PrP^Sc^ deposition at inflammatory sites is the presence of newly formed lymphoid follicles, which in the natural host species likely generate an ideal micro-environment for the scrapie agent’s replication. Interestingly, no influence on ectopic PrP^Sc^ deposition was apparently exerted by the etiologic agent, as highlighted by the examples of MVV and lungworm infections. No additional influence on ectopic PrP^Sc^ deposition was also apparently exerted by the host’s PrP genotype and the body districts developing chronic inflammation. The IHC pattern of PrP^Sc^ deposition within newly formed lymphoid follicles was similar to that reported in follicles of secondary lymphoid tissues from scrapie-affected sheep [22], [23]. Furthermore, by performing cell immunophenotyping and qRT-PCR in chronically inflamed organs harboring newly formed lymphoid follicles, we were able to observe a micro-architecture and molecular profile highly consistent with those of secondary lymphoid follicles, as previously reported [12], [19]. It is also of interest that we could immunohistochemically demonstrate the presence of a consistent FDC network inside the GCs of these newly formed follicles; all mammary glands with lymphofollicular mastitis also displayed significant overexpression of LTα and LTβ cytokines, as well as CXCL13 chemokines on behalf of B cells and mature FDCs respectively. Within both constitutive and newly formed lymphoid follicles, differentiation from stromal cells, maturation, and maintenance of FDCs are modulated by LTα and LTβ cytokines, which form a LTαβ heterotrimeric complex [24] signaling through the LTβR expressed on the FDC membrane, with this most likely representing a critical step in making these cells “permissive” to prion colonization [25]. More precisely, FDCs have been recently proven to be a very active PrP^Sc^ replication site in secondary lymphoid tissues [3]. During lymphoneogenesis, in fact, CXCL13 stimulates the chemotaxis of B lymphocytes [19] by an *ad hoc* feedback loop [26].

Overall, our data suggests that PrP^Sc^ accumulates in inflamed tissues, morphologically and molecularly mimicking constitutive secondary lymphoid tissues.

The argument that IHC is not a sufficiently sensitive method to detect minute PrP^Sc^ amounts should also be taken into consideration. Nonetheless, many studies in both natural and experimental TSE models have shown that IHC is able to detect PrP^Sc^ similarly to WB in a number of extra-neural and extra-lymphatic tissues such as skeletal muscle [4], kidney [27], [28], and skin [29]. Indeed, through IHC we could easily demonstrate the precise anatomic localization of PrP^Sc^ aggregates in newly formed follicles but not in granulomas, even when these two patterns of chronic inflammation occurred in close proximity to each other, as observed in the lung and ileum from the sheep investigated herein.

Furthermore we compared mammary glands affected by lymphofollicular mastitis to mammary glands with either lymphoproliferative/non-lymphofollicular or granulomatous mastitis. LTα and LTβ gene expression levels were significantly higher in the former, with this likely resulting in a specific signal for ectopic PrP^Sc^ deposition. We did not observe a similar behavioral pattern for TNFα, which showed consistent expression levels within both lymphofollicular and granulomatous inflammatory foci. High TNFα gene expression levels were previously reported in granulomas during tuberculosis, in the course of which TNFα is known to represent the dominant cytokine [30]. Furthermore, as far as chemokine and adhesion molecule gene expression levels are concerned, we found a significant overexpression of CXCL12 and VCAM-1 in mammary glands affected by granulomatous mastitis *versus* healthy ovine mammary glands. CXCL12, a chemokine broadly expressed by several tissues, has been shown to be a chemoattractant for naïve B and T cells, and for DCs *in vitro* [31]. We speculate that CXCL12 might play a role in the homing of B and T lymphocytes within granulomatous lesions. In this respect, we were able to determine the precise location of B and T cells in sheep granulomas by means of cell immunophenotyping.

In light of the above observations, only LTα and LTβ cytokines should be regarded as reliable triggers and predictors for ectopic PrP^Sc^ deposition within chronic inflammatory lesions affecting the ovine mammary gland. An apparently conflicting result of our work is represented by the fact that the different LTβR gene expression levels between healthy mammary glands and those affected by lymphofollicular mastitis were not statistically significant (P = 0.056), despite the significant differences observed in the expression levels of the respective ligands, LTα and LTβ. LTβR, which has pleiotropic functions, is expressed by FDCs and also by other stromal cells as well as by epithelial cells [32]. Therefore, we speculate that in mammary glands LTβR may also be expressed under physiologic, non-inflammatory conditions. Interestingly, similar results have also been reported by comparing lymphofollicular lesions from rheumatoid arthritis-affected patients with healthy control individuals [33].

Nevertheless, the overexpression of LTα and LTβ transcripts detected in the present study confirms the key role these molecular determinants play during lymphoneogenesis in a natural disease model. For the first time, there is evidence that the lymphoneogenesis process observed during MVV infection shares a common signaling pathway with that reported in the course of other infectious disease conditions affecting humans, including chronic hepatitis C [34], *Campylobacter pylori*-induced gastritis [16], and chronic Lyme disease [35].

Therefore, the mammary gland seems to provide a highly valuable tissue model for investigating the role of chemokines in the recruitment and positioning of immune cells within the specific context of inflammatory lesions’ development.

Newly formed lymphoid follicles can be differentiated from secondary ones by the lack of afferent lymphatic vessels [12]. However, it still remains to be verified whether newly formed lymphoid structures have an internal canalicular system, which is crucial for the circulation of immune cells and molecules involved in the host’s immune response [36]. The latest advances in understanding the molecular characteristics of lymphatic vessels have prompted investigators to use the lymphatic vessel endothelial hyaluronan receptor-1 (LYVE-1), which is a member of the Link protein family involved in leukocyte migration, as a reliable marker of the lymphatic canalicular system [37]. Furthermore, a recent study comparing LTα knockout mice (LTα^−/−^) and wildtype mice, showed LYVE-1 overexpression during new lymphoid tissue formation, with a direct and significant contribution of LTα but not of LTβ, with LTα_3_ signaling through the TNFR1 receptor [38].

Our results confirm that both LYVE-1 and LTα are significantly up-regulated in sheep mammary glands affected by lymphofollicular mastitis. This suggests that newly formed follicles will progressively develop an efficient lymphatic micro-vessel network, which is most likely an additional factor supporting an ideal micro-environment for PrP^Sc^ replication.

We also demonstrated different levels of PrP and PrP gene expression in healthy and inflamed ovine mammary glands. However, only PrP gene expression was significantly higher in the mammary glands affected by granulomatous mastitis. In this respect, although a previous study showed that PrP was overexpressed by both keratinocytes and infiltrating mononuclear cells in chronic inflammatory skin diseases [39], another work on experimentally scrapie-infected sheep reported that the magnitude of PrP^Sc^ accumulation does not depend on the PrP expression levels in different splenic compartments [40]. Likewise, in our study the different levels of PrP and PrP gene expression in chronically inflamed tissues were not a factor promoting ectopic PrP^Sc^ deposition.

In conclusion, our findings clearly suggest that, in sheep co-affected by scrapie and chronic inflammatory conditions such as those caused by MVV and lungworms, only newly formed lymphoid follicles, independently from their anatomical location and causative factor, provide a suitable microenvironment supporting scrapie agent’s replication, with an increased risk of prion shedding through body secretions/excretions.

## References

[1] Prusiner SB (1998) Prions. Proc Natl Acad Sci USA 95: 13363–13383. 9811807 10.1073/pnas.95.23.13363 PMC33918

[2] Andréoletti O, Berthon P, Marc D, Sarradin P, Grosclaude J, et al (2000) Early accumulation of PrP(Sc) in gut-associated lymphoid and nervous tissues of susceptible sheep from a Romanov flock with natural scrapie. J Gen Virol 81: 3115–3126. 11086143 10.1099/0022-1317-81-12-3115

[3] McCulloch L, Brown KL, Bradford BM, Hopkins J, Bailey M, et al (2011) Follicular dentritic cell-specific prion protein (PrP^Sc^) expression alone is sufficient to sustain prion infection in the spleen. PLoS Pathog 7: e1002402. 22144895 10.1371/journal.ppat.1002402 PMC3228802

[4] Andréoletti O, Simon S, Lacroux C, Morel N, Tabouret G, et al (2004) PrP^Sc^ accumulation in myocytes from sheep incubating natural scrapie. Nat Med 10: 591–593. 15156203 10.1038/nm1055

[5] Andréoletti O, Lacroux C, Chabert A, Monnereau L, Tabouret G, et al (2002) PrP(Sc) accumulation in placentas of ewes exposed to natural scrapie: influence of foetal PrP genotype and effect on ewe-to-lamb transmission. J Gen Virol 83: 2607–2616. 12237445 10.1099/0022-1317-83-10-2607

[6] Vascellari M, Nonno R, Mutinelli F, Bigolaro M, Di Bari MA, et al (2007) PrP^Sc^ in salivary glands of scrapie-affected sheep. J Virol 8: 4872–4876. 10.1128/JVI.02148-06 PMC1900156 17301130

[7] Heikenwalder M, Zeller N, Seeger H, Prinz M, Klöhn PC, et al (2005) Chronic lymphocytic inflammation specifies the organ tropism of prions. Science 307: 1107–1110. 15661974 10.1126/science.1106460

[8] Heikenwalder M, Kurrer MO, Margalith I, Kranich J, Zeller N, et al (2008) Lymphotoxin-dependent prion replication in inflammatory stromal cells of granulomas. Immunity 29: 998–1008. 19100703 10.1016/j.immuni.2008.10.014

[9] Ligios C, Sigurdson CJ, Santucciu C, Carcassola G, Manco G, et al (2005) PrPSc in mammary glands of sheep affected by scrapie and mastitis. Nat Med 11: 1137–1138. 16270061 10.1038/nm1105-1137

[10] Lacroux C, Simon S, Benestad SL, Maillet S, Mathey J, et al (2008) Prions in milk from ewes incubating natural scrapie. PLoS Pathog 4: e1000238. 19079578 10.1371/journal.ppat.1000238 PMC2587713

[11] Hamir AN, Kunkle RA, Miller JM, Hall SM (2006) Abnormal prion protein in ectopic lymphoid tissue in a kidney of an asymptomatic white-tailed deer experimentally inoculated with the agent of chronic wasting disease. Vet Pathol 43: 367–369. 16672586 10.1354/vp.43-3-367

[12] Aloisi F, Pujol-Borrell R (2006) Lymphoid neogenesis in chronic inflammatory diseases. Nat Rev Immunol 6: 205–217. 16498451 10.1038/nri1786

[13] Weninger W, Carlsen HS, Goodarzi M, Moazed F, Crowley MA, et al (2003) Naive T cell recruitment to non-lymphoid tissues: a role for endothelium-expressed CC chemokine ligand 21 in autoimmune disease and lymphoid neogenesis. J Immunol 170: 4638–4648. 12707342 10.4049/jimmunol.170.9.4638

[14] Armengol MP, Cardoso-Schmidt CB, Fernández M, Ferrer X, Pujol-Borrell R (2003) Chemokines determine local lymphoneogenesis and a reduction of circulating CXCR4+ T and CCR7 B and T lymphocytes in thyroid autoimmune diseases. J Immunol 170: 6320–6328. 12794165 10.4049/jimmunol.170.12.6320

[15] Serafini B, Rosicarelli B, Magliozzi R, Stigliano E, Aloisi F (2004) Detection of ectopic B-cell follicles with germinal centers in the meninges of patients with secondary progressive multiple sclerosis. Brain Pathol 14: 164–174. 15193029 10.1111/j.1750-3639.2004.tb00049.x PMC8095922

[16] Kobayashi M, Mitoma J, Nakamura N, Katsuyama T, Nakayama J, et al (2004) Induction of peripheral lymph node addressin in human gastric mucosa infected by *Helicobacter pylori.* . Proc Natl Acad Sci USA 101: 17807–17812. 15591109 10.1073/pnas.0407503101 PMC539746

[17] Blacklaws BA (2012) Small ruminant lentiviruses: Immunopathogenesis of visna-maedi and caprine arthritis and encephalitis virus. Comp Immunol Microbiol Infect Dis 35: 259–269. 22237012 10.1016/j.cimid.2011.12.003

[18] Aggarwal BB (2003) Signalling pathways of the TNF superfamily: a double-edged sword. Nat Rev Immunol 3: 745–756. 12949498 10.1038/nri1184

[19] Luther SA, Lopez T, Bai W, Hanahan D, Cyster JG (2000) BLC expression in pancreatic islets causes B cell recruitment and lymphotoxin-dependent lymphoid neogenesis. Immunity 12: 471–481. 10843380 10.1016/s1074-7613(00)80199-5

[20] Cheville NF (2006) Introduction to Veterinary Pathology. Edited by Cheville NF. Ames IA Blackwell Publishing. 61–98.

[21] Khader SA, Rangel-Moreno J, Fountain JJ, Martino CA, Reiley WW, et al (2009) In a murine tuberculosis model, the absence of homeostatioc chemokines delays granuloma formation and protective immunity. J Immunol 183: 8004–8014. 19933855 10.4049/jimmunol.0901937 PMC2799945

[22] van Keulen LJ, Schreuder BE, Meloen RH, Mooij-Harkes G, Vromans ME (1996) Immunohistochemical detection of prion protein in lymphoid tissues of sheep with natural scrapie. J Clin Microbiol 34: 1228–1231. 8727908 10.1128/jcm.34.5.1228-1231.1996 PMC228987

[23] González L, Martin S, Jeffrey M (2003) Distinct profiles of PrP(d) immunoreactivity in the brain of scrapie- and BSE- infected sheep: implications for differential cell targeting and PrP processing. J Gen Virol 84: 1339–1350. 12692301 10.1099/vir.0.18800-0

[24] Browning JL, Ngam-ek A, Lawton P, DeMarinis J, Tizard R (1993) Lymphotoxin beta, a novel member of the TNF family that forms a heteromeric complex with lymphotoxin on the cell surface. Cell 72: 847–856. 7916655 10.1016/0092-8674(93)90574-a

[25] Montrasio F, Frigg R, Glatzel M, Klein MA, Mackay F (2000) Impaired prion replication in spleens of mice lacking functional follicular dendritic cells. Science 288: 1257–1259. 10818004 10.1126/science.288.5469.1257

[26] Ansel KM, Ngo VN, Hyman PL, Luther SA, Förster R (2000) A chemokine-driven positive feedback loop organizes lymphoid follicles. Nature 406: 309–314. 10917533 10.1038/35018581

[27] Ligios C, Cancedda GM, Margalith I, Santucciu C, Madau L, et al (2007) Intraepithelial and interstitial deposition of pathological prion protein in kidneys of scrapie-affected sheep. PLoS One 2: e859. 17848990 10.1371/journal.pone.0000859 PMC1964536

[28] Gregori L, Kovacs GG, Alexeeva I, Budka H, Rohwer RG (2008) Excretion of transmissible spongiform encephalopathy infectivity in urine. Emerg Infect Dis 14: 1406–1412. 18760007 10.3201/eid1409.080259 PMC2603099

[29] Thomzig A, Schulz-Schaeffer W, Wrede A, Wemheuer W, Brenig B, et al (2007) Accumulation of pathological prion protein PrP^Sc^ in the skin of animals with experimental and natural scrapie. PLoS Pathog 3(5): e66. 17530923 10.1371/journal.ppat.0030066 PMC1876502

[30] Russell DG (2007) Who puts the tubercle in tuberculosis? Nat Rev Microbiol 5: 39–47. 17160001 10.1038/nrmicro1538

[31] Luther SA, Bidgol A, Hargreaves DC, Schmidt A, Xu Y, et al (2002) Differing activities of homeostatic chemokines CCL19, CCL21, and CXCL12 in lymphocytes and dendritic cell recruitment and lymphoid neogenesis. J Immunol 169: 424–433. 12077273 10.4049/jimmunol.169.1.424

[32] Browning JL (2008) Inhibition of the lymphotoxin pathway as a therapy for autoimmune disease. Immunol Rev 223: 202–220. 18613838 10.1111/j.1600-065X.2008.00633.x

[33] O’Rourke KP, O’Donoghue G, Adams C, Mulcahy H, Molloy C (2008) High levels of lymphotoxin-beta (LT-beta) gene expression in rheumatoid arthritis synovium: clinical and cytokine correlations. Rheumatol Int 28: 979–986. 18379788 10.1007/s00296-008-0574-z

[34] Murakami J, Shimizu Y, Kashii Y, Kato T, Minemura M, et al (1999) Functional B-cell response in intrahepatic lymphoid follicles in chronic hepatitis C. Hepatology. 30: 143–150. 10.1002/hep.510300107 10385650

[35] Ghosh S, Steere AC, Stollar BD, Huber BT (2005) *In situ* diversification of the antibody repertoire in chronic Lyme arthritis synovium. J Immunol 174: 2860–2869. 15728496 10.4049/jimmunol.174.5.2860

[36] von Andrian UH, Mempel TR (2003) Homing and cellular traffic in lymph nodes. Nat Rev Immunol 3: 867–878. 14668803 10.1038/nri1222

[37] Jackson DG (2004) Biology of the lymphatic marker LYVE-1 and applications in research into lymphatic trafficking and lymphangiogenesis. APMIS 112: 526–538. 15563314 10.1111/j.1600-0463.2004.apm11207-0811.x

[38] Mounzer RH, Svendsen OS, Baluk P, Bergman CM, Padera TP (2010) Lymphotoxin-alpha contributes to lymphangiogenesis. Blood 116: 2173–2182. 20566898 10.1182/blood-2009-12-256065 PMC2951858

[39] Pammer J, Weninger W, Erwin Tschachler (1998) Human keratinocytes express cellular prion-related protein *in vitro* and during inflammatory diseases. Am J Pathol 153: 1353–1358. 9811324 10.1016/S0002-9440(10)65720-3 PMC1853387

[40] Sørby R Austbø, Press CM, Skretting G, Landsverk T, et al (2009) PrP expression, PrP^Sc^ accumulation and innervation of splenic compartments in sheep experimentally infected with scrapie. Plos One 4(9): e6885. 19727393 10.1371/journal.pone.0006885 PMC2731221

